# Bleomycin-Induced Cryptogenic Organizing Pneumonia Manifested as Spontaneous Pneumothorax in a Patient with Classic Seminoma

**Published:** 2019-03

**Authors:** Diana Lizbeth Ortíz-Farías, Stephanie López-Romero, Hirian Alonso Moshe Barrera-Pérez, Gary Kosai Vargas-Mendoza, Arturo Cortes-Telles

**Affiliations:** 1 Departamento de Neumología y Cirugía de Tórax. Hospital Regional de Alta Especialidad de la Península de Yucatán.; 2 Anatomía Patológica ANAPAT. Mérida, Yucatán, México

**Keywords:** Bleomycin, Cryptogenic Organizing Pneumonia, Pneumothorax, Toxicity

## Abstract

Cryptogenic Organizing Pneumonia (COP) can manifest like a collagen disorder or infectious diseases, or be caused by drug induced toxicity. This paper presents the case of a 24 year-old man diagnosed with classic seminoma, treated with chemotherapy scheme that included bleomycin (accumulated dose, 120 units). The patient was admitted at the hospital due to rapidly-progressing dyspnea and thoracic pain. The diagnostic approach revealed the presence of a spontaneous pneumothorax, while a lung biopsy documented COP. Despite treatment, the patient died from disease progression.

## INTRODUCTION

Pulmonary toxicity caused by bleomycin is the most prevalent and best-defined pulmonary disease induced by chemotherapy. The clinical expression includes symptoms that may vary in intensity, including dyspnea, cough, thoracic pain and crackles during physical auscultation. Also, it is common to detect the presence of opacities on chest x-rays (1). Some reports have found the unusual but clinically-significant presence of pulmonary lesions induced by bleomycin that have a nodular appearance and may simulate metastasis of the primary tumor. When this disjunctive appears, it is advisable to perform a pulmonary biopsy in order to determine toxicity due to bleomycin, or evidence of distal implants from the primary tumor (2).

The objective of this report is to present the case of a male patient in his 20s diagnosed with classic seminoma who was undergoing chemotherapy treatment based on bleomycin. He arrived at the Emergency Department for evaluation of a rapidly-progressing dyspnea and thoracic pain, secondary to a spontaneous pneumothorax. The assessments concluded that the alterations were precipitated by the presence of Cryptogenic Organizing Pneumonia (COP) secondary to the bleomycin use.

## CASE SUMMARIES

The case was a 24-year-old male with history of smoking (tobacco index of 2.7 packs/year), diagnosis of classic seminoma (T2N3M0) S3, undergoing treatment with a chemotherapy scheme based on cisplatin, etoposide and bleomycin (30 units/session). He had completed 4 cycles of chemotherapy.

His symptoms began 3 days before he was admitted to the Emergency Department complaining of productive cough, mild-to-moderate dyspnea; 48 hours later, pleuritic pain appeared in the left hemithorax. Physical examination of the thorax revealed no respiratory sound and tympanism in the left hemithorax. Chest X-rays confirmed a left pneumothorax. An endopleural tube was placed without complications.

**Figure 1.**
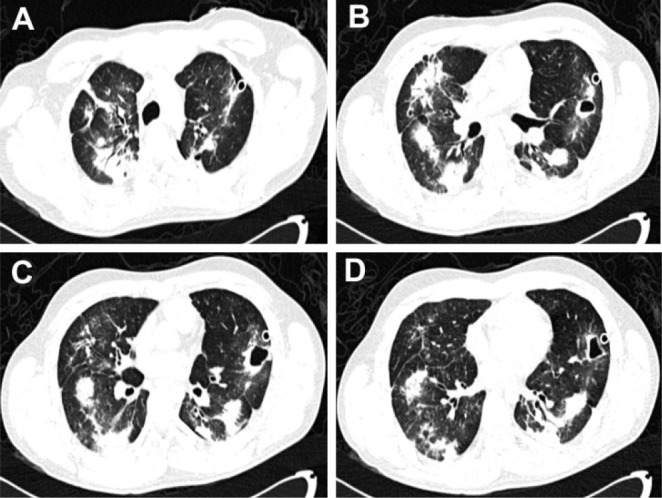
Chest CT - Scan of patient.

**Figure 2.**
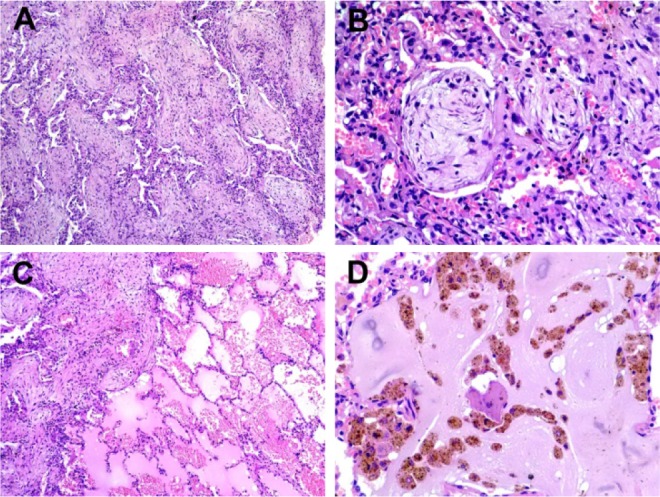
Histologic examination of biopsy specimen.

During follow-up, a chest CT scan revealed bilateral interstitial septal thickening, cysts in the left upper and lower lobes, tractions bronchiectasis, and consolidation areas with predominance in both lower lobes (Figure 1). A video thoracoscopy-guided biopsy was performed; histologic examination was consistent with COP (Figure 2). Prednisone dose (0.75 mg/kg/day), and oxygen therapy was initiated, but there were no signs of clinical response after 10 days. The patient developed an acute worsening; intubation was required with invasive mechanical ventilation, and despite ventilatory support, the patient died.

## DISCUSSION

To the best of our knowledge, this is the first case to integrally document the existence of COP secondary to pulmonary toxicity induced by bleomycin in a patient with a germ tumor of the classical seminoma type. Also, our extensive research of the available literature suggests that this is the first report of a case of COP expressed as a spontaneous pneumothorax.

Bleomycin is an antitumor antibiotic that was isolated from a strain of *Streptomyces verticillus* in 1966. Despite the development of new drugs, it continues to be used as an important element in certain chemotherapy schemes for curable diseases, including germ cells tumors and Hodgkin’s lymphoma (3).

Pulmonary toxicity induced by bleomycin is a clearly-recognized secondary effect. Several articles have reported a prevalence of 40–45% in patients treated with schemes that include bleomycin with mortality resulting in 1–3% of all cases (4). Age, the accumulated dose of the drug, kidney function, the severity of the underlying oncological problem at the time of presentation, and the concomitant use of oxygen, radiotherapy, other chemotherapeutic agents, and stimulating factors of hematopoietic colonies can all influence the risk of developing bleomycin-induced pulmonary toxicity (5). Accumulated doses above 400 units are associated with higher odds of pulmonary toxicity, though lesions have occurred at doses below 100 units (1). The patient’s clinical history showed an accumulated dose of 120 units. Clearly this is below the upper risk level; it does fall within the range of reports that have documented toxicity. The physiopathology of toxicity due to bleomycin is attributable to endothelial damage induced in the pulmonary vasculature which, when added to the harmful effects mediated by radicals with cytokines and oxygen, stimulated a cascade of inflammatory markers, activation of fibroblasts and collagen deposition. Bleomycin hydrolase is activated in all tissues, except the skin and lungs, which may explain the specific toxicity of this particular drug for these organs (6, 7). Several pulmonary syndromes have been related to the use of bleomycin, including eosinophilic hypersensitivity, COP and, at even higher frequencies, hypersensitivity pneumonitis that, in later stages may progress to fibrosis (8).

COP is a form of diffuse idiopathic interstitial pulmonary disease. It is confirmed by pathology and, in some cases, may be secondary to an identifiable etiology (viral infection, toxic gases, medications, gastroesophageal reflux, radiotherapy, connective tissue disorders) (9, 10). Clinical manifestations begin with a mild flu-like illness, fever, cough, progressive dyspnea, general malaise, anorexia, and weight loss. In isolated cases, dyspnea may be severe, especially in the presence of a rapidly-progressing disease. The development of pneumothorax is very rare; in fact, to date, the literature contains no reported cases of pneumothorax as a cause of primary attention in patients with bleomycin-induced COP (11, 12). Our review of published cases found no reports in which the initial presentation was related to this finding (1, 4).

While the physiopathology of pneumothorax associated with neoplasia is not completely unknown, the mechanisms that have been postulated include dynamic alterations of the peripheral airway due to invasion by the cancer, which acts as a retention valve that generates air entrapment and dilatation of the distal alveolar spaces with a high risk of eventual rupture. Another mechanism proposed emerged from the development of a bronchopleural fistula caused by the interaction of the direct invasion of the tumor, necrosis of the tumor, or spontaneous intratumor vascular occlusion. In these scenarios, the rate of recurrence of pneumothorax tends to be higher compared to primary spontaneous pneumothorax (up to 80% of cases) (13, 14). Specifically, the development of pneumothorax as a complication of chemotherapy in cases of metastatic germ cell tumors has been reported only sporadically. In an exhaustive study, Smevik and Klepp failed to identify a single case of secondary pneumothorax in 79 patients with histories of disseminated testicular cancer (14). Finally, we have no knowledge of any report that has attempted to define the optimal pharmacological strategy for treating COP patients. Current treatment regimens are based on patterns of consensus. The initial clinical presentation, disease severity, and the degree of response must all be taken into account when determining the appropriate treatment regimens and duration of therapy. The vast majority of patients are treated with oral glucocorticoids, which noticeably improve the symptoms. Current recommendations advise beginning with prednisone at a dose of 0.75–1 mg/kg per day, then freeing from this drug for 6–12 months (10, 12). Although our patient was managed in accordance with, and received treatment based on these recommendations, he died.

## CONCLUSION

We have discussed the first case that debuted with pneumothorax secondary to COP caused by bleomycin-induced pulmonary toxicity. Cases with a history of classic seminoma during chemotherapy that applies bleomycin must be strictly-supervised and accompanied by follow-up measures that include imagenology and quantification of the accumulated dose of bleomycin. Also, we suggest monitoring lung function (spirometry and monoxide diffusing capacity) in order to opportunely identify any abnormalities that would indicate the need for complementary treatments to reduce the risk of disease progression with potentially fatal repercussions.
